# A conserved domain of *Cfap298* governs left–right symmetry breaking in vertebrates

**DOI:** 10.1242/jcs.264129

**Published:** 2025-10-31

**Authors:** Marvin Cortez, Cullen B. Young, Katherine A. Little, Daniel T. Grimes, Danelle Devenport, Rebecca D. Burdine

**Affiliations:** Department of Molecular Biology, Princeton University, Princeton, NJ 08544, USA

**Keywords:** Cfap298, Cilia, Left–right patterning, Zebrafish, Mouse

## Abstract

*Cfap298* is a highly conserved gene required for ciliary motility and dynein arm assembly, with known roles in left–right (LR) patterning in zebrafish and links to human ciliopathies. Here, we describe a *Cfap298* mutant allele, *Cfap298^ΔΔS^*, which selectively disrupts LR axis establishment in mice. Mutant embryos display organ laterality defects and abnormal *Nodal*, *Pitx2* and *Lefty1* expression, consistent with an early disruption in LR symmetry breaking. LR asymmetry is established by leftward fluid flow in the node, generated by planar-polarized cilia. Although *cfap298* mutations are reported to affect planar polarity, we did not observe changes in cilia position, length or CELSR1 localization within the node, suggesting that *Cfap298^ΔΔS^* functions at the level of cilia motility. Accordingly, cilia lining the trachea of *Cfap298^ΔΔS^* mutants fail to beat or beat incorrectly. Expression of the *Cfap298^ΔΔS^* variant in zebrafish partially rescues body curvature defects but fails to rescue LR defects of *cfap298* (*kurly*) loss-of-function mutants. These results confirm a conserved role for *Cfap298* in mammalian LR patterning and identify a previously unreported region of CFAP298 with a conserved and essential role in cilia motility.

## Teaser

**Summary:** A previously unreported *Cfap298* mutation disrupts left–right patterning in mice by impairing ciliary motility, revealing a conserved protein region essential for this function across species.

## INTRODUCTION

Motile cilia are microtubule-based structures used by many organisms throughout the animal kingdom to move fluids or generate flow. In mammals, motile cilia line the trachea to clear the airways while motile cilia on ependymal cells propel cerebral spinal fluid throughout brain ventricles. Motile cilia in the oviduct generate flow to move oocytes towards the uterus and form the flagella that propel sperm towards the oocyte (Spassky and Meunier, 2017; Hilgendorf et al., 2024). Dysregulation of motile cilia function often leads to the development of congenital disorders collectively known as ciliopathies (Spassky and Meunier, 2017; Hilgendorf et al., 2024).

Motile cilia also play crucial roles in establishing the left–right (LR) body axis during development in humans, mice, *Xenopus* and zebrafish. Motile cilia in structures collectively known as left–right organizers (LROs) generate fluid flows that culminate in the asymmetric expression of the TGFβ ligand Nodal on the left in the lateral plate mesoderm (LPM) (Nonaka et al., 2002; Grimes and Burdine, 2017; Blum and Ott, 2019; Little and Norris, 2021; Forrest et al., 2022). The mouse LRO is the node, a transient pit-like embryonic structure with planar polarized motile cilia that move fluid across the node to the left side. Crown cells surrounding the node are proposed to sense the leftward fluid flow using immotile primary cilia (Grimes and Burdine, 2017; Hashimoto et al., 2010). In zebrafish, the LRO is Kupffer's vesicle, a transient fluid-filled epithelial sac where motile cilia generate a counterclockwise flow. Mutations in cilia motility genes perturb fluid flow in LROs disrupting left-sided *Nodal* expression, resulting in abnormal organ positioning within the body cavity known as *situs inversus* (mirror image arrangement) or heterotaxy (random arrangements) (Grimes and Burdine, 2017).

The cilia and flagella-associated protein 298 (CFAP298) is essential for cilia motility in multiple species (Austin-Tse et al., 2013; Jaffe et al., 2016). CFAP298 is required in the cytoplasm for proper preassembly of axonemal dyneins needed for cilia motility and, thus, LR patterning (Wang et al., 2022; Jaffe et al., 2016; Austin-Tse et al., 2013). Mutations in the zebrafish ortholog *kurly* lead to loss of inner and outer dynein arms within the ciliary axoneme, resulting in immotile cilia and LR patterning defects (Jaffe et al., 2016). Furthermore, variants in human CFAP298 have been identified in individuals with heterotaxia (Austin-Tse et al., 2013). CFAP298 may also play a role in planar cell polarity (PCP), as *kurly* mutants in zebrafish display cilia polarity defects in the kidney and knockdown of CFAP298 causes loss of asymmetric PCP protein localization in *Xenopus* larval skin cells (Jaffe et al., 2016). However, the role of CFAP298 in planar polarity has not been further elucidated.

Utilizing CRISPR-Cas9, we generated a mutation, *Cfap298^ΔΔS^* that alters three amino acids in a highly conserved region in the mouse ortholog of *Cfap298. Cfap298^ΔΔS^* embryos display *situs inversus* and heterotaxia accompanied by abnormal expression of *Nodal*, *Pitx2* and *Lefty1*. We show *Cfap298^ΔΔS^* specifically affects cilia motility and not planar cell polarity in the mouse node. Rescue experiments in zebrafish demonstrate that, although *Cfap298^ΔΔS^* mRNA retains some function, it is unable to rescue LR defects in *cfap298* mutant zebrafish embryos. Thus, the *Cfap298^ΔΔS^* allele uncovers a three amino acid region essential for the cilia motility-related functions of CFAP298.

## RESULTS

### *Cfap298^ΔΔS^* mutants display defects in organ laterality

**Fig. 1.**
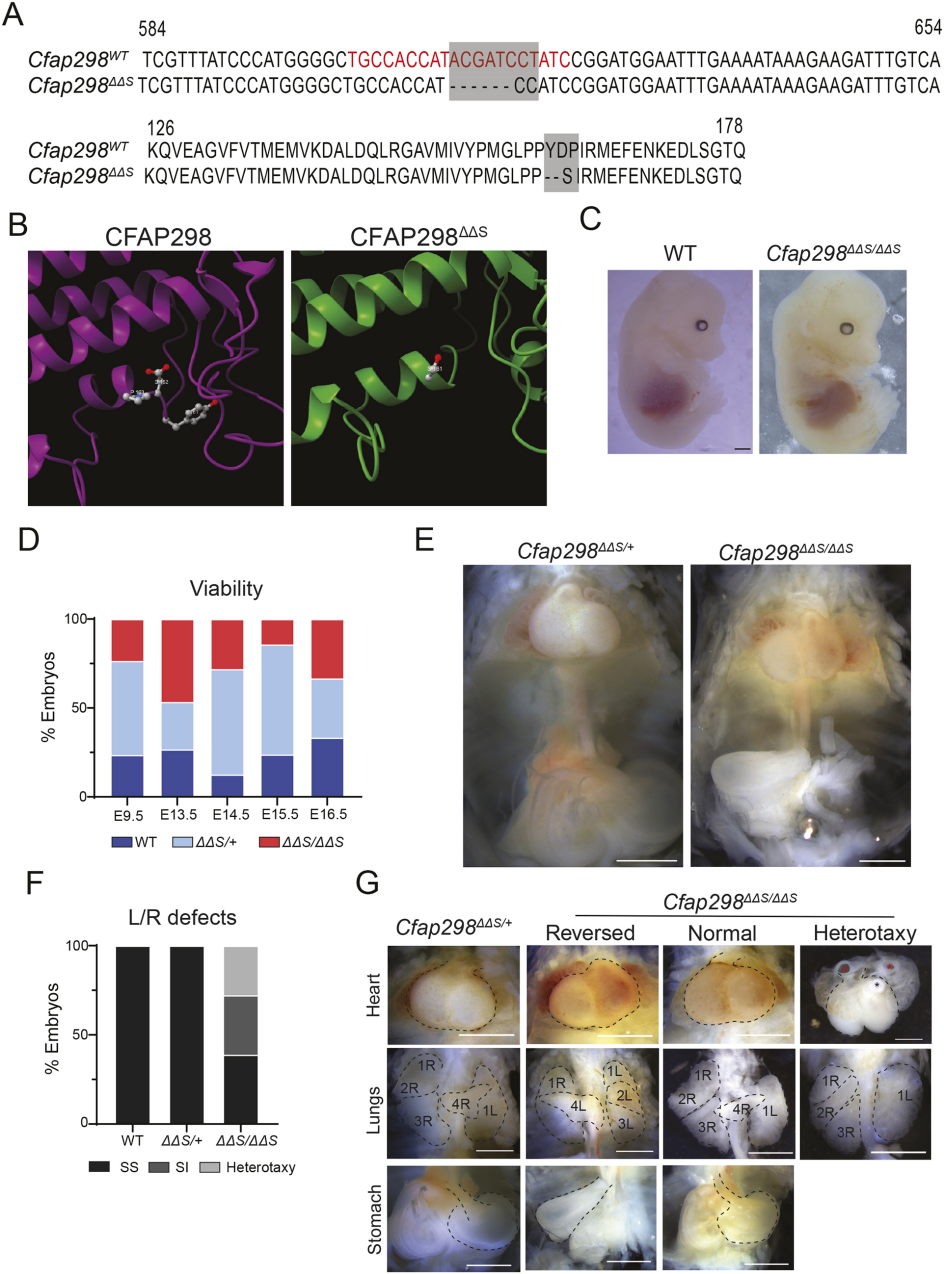
***Cfap298^ΔΔS^* mutants display left–right laterality defects.** (A) Generation of the *Cfap298^ΔΔS^* mutant allele by targeting exon 4 of the *Cfap298* gene using CRISPR/Cas9 gene targeting. Guide recognition sequence within the exon 4 sequence is in red on the wild-type sequence. The *Cfap298^ΔΔS^* mutation consists of a 6 base deletion and a base substitution of T→C, resulting in the removal of two amino acids at positions 161 an 162, and a missense mutation of P163S (gray shading). (B) AlphaFold predicted structures of CFAP298 and CFAP298^ΔΔS^ proteins in the region of the *ΔΔS* mutation. Y161 and D162 are labeled in wild-type CFAP298, and S161 is labeled on CFAP298^ΔΔS^ (Abramson et al., 2024). (C) Whole-embryo images of E14.5 wild-type and *Cfap298^ΔΔS^*^/*ΔΔS*^ embryos. Scale bar: 1 mm. (D) Viability distribution of wild-type, heterozygous and homozygous mutant embryos at E9.5 (*n*=17), E13.5 (*n*=15), E14.5 (*n*=33), E15.5 (*n*=21) and E16.5 (*n*=6). (E) Representative images of body cavities showing heart, lungs and stomach positions from *Cfap298^ΔΔS^*^/+^ and *Cfap298^ΔΔS^*^/*ΔΔS*^ E14.5 embryos. Scale bars: 1 mm. (F) Quantification of *situs solitus*, *situs inversus* and heterotaxy from wild-type (*n*=21), *Cfap298^ΔΔS^*/+ (*n*=46) and *Cfap298^ΔΔS^*^/*ΔΔS*^ (*n*=18) embryos from E13.5–E15.5 stages. (G) Representative images of heart, lung and stomach from E14.5 *Cfap298^ΔΔS^*^/+^ and *Cfap298^ΔΔS^*^/*ΔΔS*^ embryos displaying with *situs solitus* (normal), *situs inversus* (reversed) and heterotaxy phenotypes. Hearts and stomachs are outlined. Individual lung lobes are outlined and labeled with their position on either left (L) or right (R). Atria (red asterisks) and ventricle (black asterisk) are indicated in the heart with heterotaxy. Scale bars: 1 mm.

*Cfap298* (*C21ORF59*; *kurly*; *FBB18*) encodes a small 290 amino acid protein that likely contains a ubiquitin-like (UBL) domain and loop domain based on a recent crystal structure of the *Chlamydomonas* ortholog FBB18 (Yamamoto et al., 2025). To investigate the functional role of *Cfap298* in mouse development, we isolated a previously unreported allele, *Cfap298^ΔΔS^*, using CRISPR-Cas9 genome editing. The allele contains a six-nucleotide deletion together with two nucleotide substitutions in exon 4, resulting in the deletion of two amino acids (Y161 and D162) and a missense mutation (P163S) at an adjacent residue (Fig. 1A). The three affected amino acids map to an alpha helix adjacent to the loop domain and immediately before the UBL domain (Fig. 1B; Fig. S1B). Sequence conservation analysis using DMfold (deepMSA2; Zheng et al., 2024) identified residues D162 and P163 as evolutionarily conserved (Fig. S1A).

To examine the developmental consequences of this mutation, we analyzed embryos at embryonic day 14.5 (E14.5). Homozygous mutants (*Cfap298^ΔΔS/ΔΔS^*) displayed no overt morphological abnormalities externally and were recovered at expected Mendelian ratios, indicating that embryonic viability at mid-gestation was unaffected (Fig. 1C,D). Given the established role of *Cfap298* orthologs in LR symmetry breaking (Austin-Tse et al., 2013; Jaffe et al., 2016), we assessed internal organ situs from E13.5 to E15.5. Whereas wild-type and *Cfap298^ΔΔS/+^* heterozygous embryos exhibited exclusively *situs solitus*, homozygous mutants displayed a significant incidence of LR patterning defects (60%, 11/18 embryos), including complete *situs inversus* (Fig. 1E–G; Table 1). Despite the high incidence of LR defects, we were able to raise one *Cfap298^ΔΔS^* mutant to adulthood. This mutant displayed heterotaxy, including a reversed heart, right mirrored liver, left sided stomach and left lung isomerism compared to a *Cfap298^ΔΔS^*^/+^ adult that displayed normal situs of these organs (Fig. S2B). These findings align closely with the previously reported phenotypes of zebrafish *kurly* mutants, which similarly display randomized visceral organ placement due to impaired motile cilia function in Kupffer's vesicle (Jaffe et al., 2016). Consistent with LR abnormalities, we observed congenital heart defects (CHDs) in *Cfap298^ΔΔS^* homozygous mutant embryos, including transposition of the great arteries (TGA), double outlet right ventricle (DORV) and pulmonary stenosis (Fig. 1G). To determine how the *Cfap298^ΔΔS^* mutation affects the CFAP298 protein, we performed western blots on E15.5 tissue lysates. Using anti-CFAP298 antibodies a ∼33 kDa band was detected in lysates from both wild-type and *Cfap298^ΔΔS/ΔΔS^* embryos. However, the amount of protein in the 33 kDa band was reduced in *Cfap298^ΔΔS/ΔΔS^* lysates compared to wild-type controls (Fig. S1C), indicating that, although the CFAP298^ΔΔS^ protein is made, it may be less stable and therefore less abundant than the wild-type protein.

**Table 1.** Stomach, heart and lung situs *Cfap298^ΔΔS^* mutant embryos

Embryo number	Genotype	Stomach situs	Heart situs	Lung situs
1	Mutant	Normal	Reversed	Abnormal
2	Mutant	Normal	L-TGA	Normal
4	Mutant	Reversed	Reversed	Reverse
5	Mutant	Normal	Normal	Normal
5	Mutant	Reversed	Reversed	Reversed
8	Mutant	Reverse	Reversed	Reversed

Representative multi-organ situs observed in embryos. *Situs solitus, situs inversus* and heterotaxy were determined by characterizing stomach, heart and lung situs. Normal stomach situs was determined as being oriented towards the left side of the body and reversed was determined as being oriented towards the right side. Normal heart situs was determined by orientation of the heart apex towards the left side of the body and reversed was determined as being oriented to the ride side. Normal lung situs was determined as the left side of the lung having one lobe, and the right side having four lobes; reversed lung situs was determined as have four lobes on the left side and one lobe on the right. In one instance, a heart was observed to be left sided but also observed to have TGA. In one instance, an embryo had an abnormal lung with three lobes on the right side and one lobe on the left.

### *Cfap298^ΔΔS^* mutants fail to establish correct expression of LR patterning genes

**Fig. 2.**
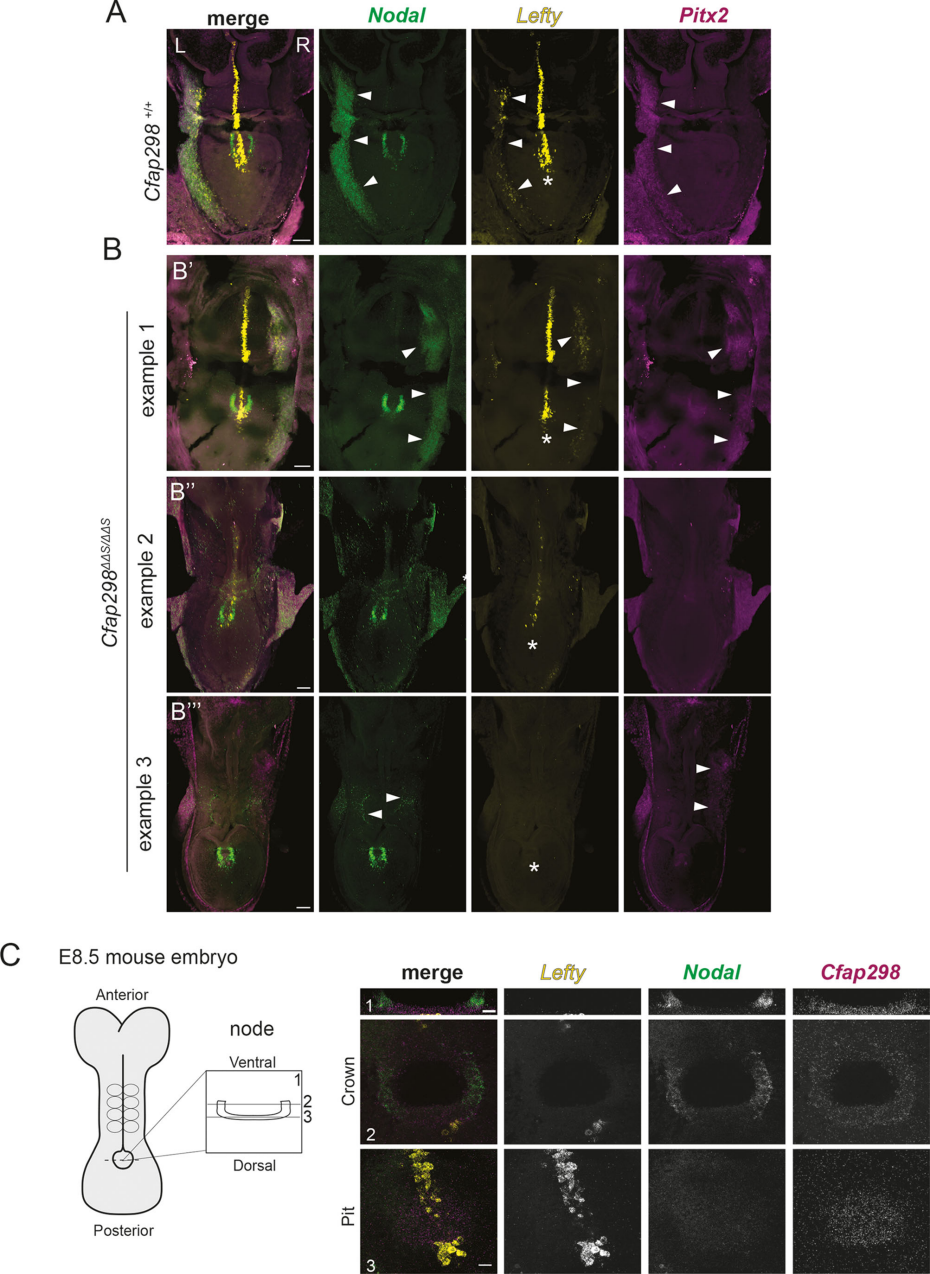
***Cfap298^ΔΔS^* mutants have perturbed left–right patterning.** (A–B‴) Representative HCR images for *Nodal* (green), *Pitx2* (magenta) and *Lefty1* (yellow) expression in a whole-mount E8.5 wild-type embryo (A) and *Cfap298^ΔΔS^* mutants (B′–B‴). Wild-type embryos exhibit left-sided *Nodal* and *Pitx2* expression in the lateral plate mesoderm (LPM) (arrowheads) with *Lefty1* expression along the embryonic midline (asterisk). (B–B‴) Images from *Cfap298^ΔΔS^* mutants showing one example of right-sided *Nodal*, *Lefty* and *Pitx2* expression along the LPM (arrowheads) (B′), an example where *Nodal* and *Pitx2* are absent in the LPM and *Lefty1* is expressed weakly at the midline (B″, asterisk), and an example of abnormal expression with reduced and bilateral *Nodal*, right-sided *Pitx2* (arrowheads) and absent *Lefty1* in the midline (asterisk) (B‴). Scale bars: 100 μm. (C) Diagram of an E8.5 wild-type embryo viewed from the dorsal side, anterior is up. Expanded view of a cross-section through the node is shown on the right. Representative images of E8.5 node labeled by HCR for *Nodal* (green), *Lefty1* (yellow) and *Cfap298* (magenta) transcripts. Cross-section (1) and planar (2 and 3) views of the node showing *Nodal* expression in the crown cells but not at the pit cells. *Lefty1*-expressing cells mark the floorplate of the neural tube are visible at the base of the pit of the node. *Cfap298*-expressing cells are visible throughout the node. Scale bars: 10 μm.

The appearance of LR defects in organ laterality is indicative of perturbed LR patterning earlier in embryogenesis. Thus, we next assessed the asymmetric expression of key genes involved in executing the LR laterality program, including *Nodal* and its target *Pitx2* in the lateral plate mesoderm (LPM) of E8.5 embryos. Spatial expression analysis by hybridization chain reaction (HCR) using probes against *Nodal*, *Pitx2* and *Lefty1* revealed left-sided *Nodal*, *Pitx2* and *Lefty1* in the LPM, and *Lefty1* expression in the midline in wild-type embryos, as expected (Fig. 2A). By contrast, *Cfap298^ΔΔS^* homozygous mutant embryos exhibited disrupted *Nodal*, *Pitx2* and *Lefty1* expression (Fig. 2B). In one example, we observed right-sided expression of *Nodal* and *Pitx2* in the LPM (Fig. 2B′). In another, *Nodal* and *Pitx2* expression was lacking in the LPM, but weak expression of *Lefty1* at the midline was present (Fig. 2B″). In another example, we observed weakened bilateral *Nodal* together with right-sided expression of *Pitx2* in the LPM and a loss of *Lefty1* at the midline (Fig. 2B‴). We conclude from these data that the LR organ laterality defects observed in E14.5 *Cfap298^ΔΔS^* mutant embryos are a result of the failure to correctly establish the LR axis.

### Node morphogenesis and cilia assembly are unaffected in *Cfap298^ΔΔS^* mutants

LR patterning requires an intact LRO, motile cilia and proper planar polarity to align cilia. Single-cell RNA sequencing (scRNA-seq) datasets from E8.5 mouse embryos (Pijuan-Sala et al., 2019) revealed that, whereas *Cfap298* transcripts are ubiquitously expressed, they are highly enriched within the node/notochord cell population, similar to the LR patterning gene *Nodal*, and consistent with expression patterns previously observed for zebrafish *kurly* (Jaffe et al., 2016; Fig. S3). Using HCR, we confirmed the widespread expression of *Cfap298* transcripts, as well as elevated levels specifically within crown and pit cells of the embryonic node at E8.5 (Fig. 2C). The conserved expression pattern of *Cfap298* in the node suggests an essential and early role in LR symmetry breaking.

**Fig. 3.**
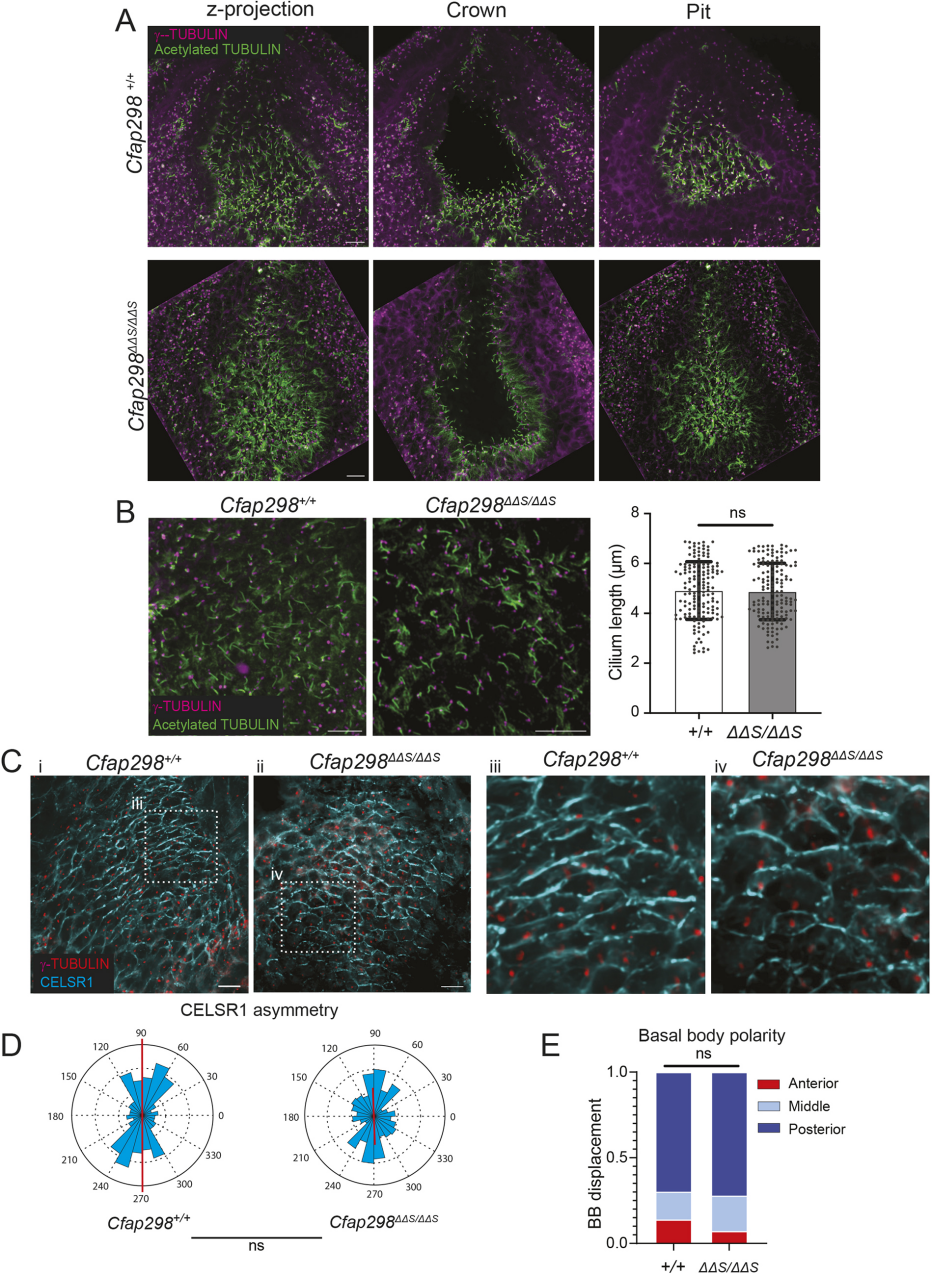
***Cfap298^ΔΔS^* mutants develop normal nodes.** (A) Representative immunofluorescent images of E8.5 nodes from wild-type and *Cfap298^ΔΔS^* mutant embryos. Embryos are stained for cilia with acetylated tubulin (green) and γ-tubulin (magenta) antibodies. For wild-type and mutant nodes, one plane is shown for the crown cells and another plane is shown for pit cells along with a *z*-projection of the whole node. (B) Images of cilia are shown for wild-type and mutant nodes. Quantifications of cilium length for wild-type (*n*=3 nodes, 150 cilia total) and mutant (*n*=3 nodes, 150 cilia total) embryos showing no significant difference (*P*=0.76 by unpaired *t*-test). (C) Images showing CELSR1 (cyan) and γ-tubulin (red) marking basal bodies in wild-type (+/+) and *Cfap298^ΔΔS^* mutant embryos. Boxes in i and ii indicate regions shown in images iii and iv, respectively. (D) Circular histograms display magnitude and orientation of CELSR1 polarity along the AP axis of wild-type (*n*=3 nodes, 99 cells total) and mutant (*n*=3, 218 cells total) embryos. Average polarity magnitudes from wild-type and mutant nodes were determined not to be significantly different (*P*=0.0691 by unpaired *t*-test). (E) Quantification of basal body polarity along the AP axis of the wild-type (*n*=3, 86 cells total) and mutant (*n*=3, 97 cells total) embryos showing no significant difference (*P*=0.2877 by Chi-squared test). ns, not significant (*P*>0.05). Scale bars: 10 μm.

To determine the cause of LR patterning defects in *Cfap298^ΔΔS^* mutants, we investigated the formation of the node and cilia. The node is a transient, pit-like structure located at the posterior midline of E8.0–E8.5 embryos. It is lined with motile cilia that generate a leftward fluid flow, initiating the symmetry-breaking event in establishment of the LR axis (Hamada, 2020). At E8.5, the nodes of *Cfap298^ΔΔS^* mutant embryos appeared morphologically normal as their overall shapes were similar to wild-type littermates (Fig. 3A). To determine whether cilia assembly was altered in *Cfap298^ΔΔS^* mutants, we measured the length of nodal cilia labeled with antibodies against acetylated tubulin but did not observe an appreciable difference in cilia length between wild-type and mutant embryos (Fig. 3B).

### *Cfap298^ΔΔS^* does not disrupt PCP establishment

In multiciliated cells of *Xenopus* epidermis, *Cfap298* mutations disrupt the asymmetric localization of the core PCP component Prickle (Pk) (Jaffe et al., 2016), suggesting that *Cfap298* may function in PCP establishment. To determine whether the *Cfap298^ΔΔS^* mutation affects PCP, we first examined planar polarity establishment in the developing epidermis, a well-established system for investigating PCP function in mouse embryos (Devenport and Fuchs, 2008; Aw et al., 2016). Although mouse skin does not contain cells with motile cilia, *Cfap298* is broadly expressed in most cell types of the skin at E14.5, including epidermal cells, hair placodes, dermal fibroblasts and the dermal condensate (Fig. S4A) (Sennett et al., 2015; Rezza et al., 2016). Thus, the skin provides an ideal system to evaluate the role of *Cfap298* in PCP without the confounding effects of cilia-driven fluid flow, which can orient and align planar polarity through positive feedback (Mitchell et al., 2007; Guirao et al., 2010). We therefore measured the asymmetric localization of the core PCP protein CELSR1 in basal cells of the epidermis, as well as the polarization and alignment of hair follicles along the anterior–posterior (AP) axis, which is the downstream output of PCP asymmetry in mammalian skin (Devenport and Fuchs, 2008). However, we did not observe a reduction in the asymmetry of CELSR1, which was enriched along the AP junctions of basal epidermal cells in both wild-type control and *Cfap298^ΔΔS^* mutant embryos (Fig. S4B). Moreover, we did not observe defects in hair follicle polarity or alignment (Fig. S4C), indicating that PCP establishment occurs normally in the epidermis of *Cfap298^ΔΔS^* mutants. Since *Cfap298* has been implicated in planar polarization of motile cilia in other organisms (Jaffe et al., 2016), we investigated the impact of the *Cfap298^ΔΔS^* mutation on PCP establishment and polarized basal body positioning within the node. Components of the PCP pathway, including VANGLl1, VANGLl2, DVL2/3, PK2 and CELSR1, are asymmetrically localized along the AP axis of the node, where they govern the posterior displacement of the basal body, which is important for leftward fluid flow (Hashimoto et al., 2010; Antic et al., 2010; Mahaffey et al., 2013; Minegishi et al., 2017). Defects in PCP within the node affect cilia placement and fluid flow, leading to defects in LR patterning. To determine whether PCP is established correctly in the nodes of *Cfap298^ΔΔS^* mutants, we measured the orientation and magnitude of CELSR1 asymmetry (Aw et al., 2016). In wild-type embryos, CELSR1 was enriched along the AP junctions of node epithelial cells, as expected. In *Cfap298^ΔΔS^* mutant embryos, CELSR1 was similarly asymmetrically polarized along the AP junctions and average polarity magnitudes between wild-type and *Cfap298^ΔΔS^* mutant nodes were not significantly different (Fig. 3C,D). Downstream of PCP establishment in the node, basal bodies become asymmetrically positioned toward the posterior of each cell (Hashimoto et al., 2010). We therefore measured the relative distance of basal bodies, marked by γ-tubulin, along the AP axis of each cell. In wild-type embryos, basal bodies were positioned toward the posterior end of pit cells, as expected. In *Cfap298^ΔΔS^* mutants, the posterior displacement of basal bodies was not significantly different compared to wild-type embryos (Fig. 3E). Collectively, these observations indicate that node morphogenesis and planar polarization occur normally in *Cfap298^ΔΔS^* mutants, and that LR defects in mutant embryos likely occur downstream or independent of cilia axoneme assembly or planar polarity.

### Cilia are immotile in *Cfap298^ΔΔS^* mutants

Given the lack of PCP defects in the node, we hypothesized that the ΔΔS mutation may impact the cilia motility function of CFAP298. To test this, we imaged the trachea, which is lined with multiciliated cells (MCCs) in *Cfap298^ΔΔS^*^/+^ control and *Cfap298^ΔΔS^* mutant adult mice. The adult mutant displayed mild hydrocephalus, possibly indicative of impaired cilia motility in the brain (Spassky and Meunier, 2017; Fig. S2A). We observed MCCs in both *Cfap298^ΔΔS/+^* and *Cfap298^ΔΔS^* mutant tracheas (Movies 1 and 2). Whereas MCCs in the *Cfap298^ΔΔS^*^/+^ trachea were highly motile, we observed severely reduced to no cilia movement in the mutant trachea (Movies 1 and 2). This shows that the *Cfap298^ΔΔS^* mutation disrupts cilia motility.

### *Cfap298^ΔΔS^* does not rescue LR defects in zebrafish

**Fig. 4.**
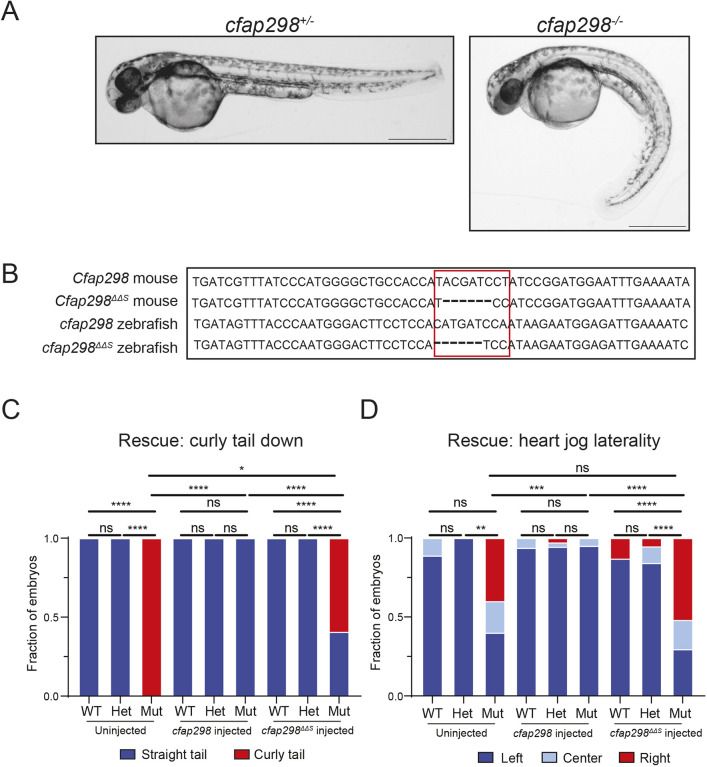
***Cfap298^ΔΔS^* does not rescue LR defects in zebrafish.** (A) Representative images of *cfap298*^+/−^ and *cfap298*^−/−^ zebrafish embryos at 48 hpf, with *cfap298*^+/−^ embryos displaying straight bodies and/or tails compared to *cfap298*^−/−^, which display curved bodies and/or tails. (B) Comparison of mouse wild-type *Cfap298* and *Cfap298^ΔΔS^* with wild-type zebrafish *cfap298* and engineered *cfap298^ΔΔS^* sequence. Area outlined in red indicates location of the *cfap298^ΔΔS^* mutation in mouse and zebrafish. (C,D) Quantification of body curvature (C) and heart position (D) observed in uninjected and injected embryos. Uninjected wild-type (*n*=9) and *cfap298*^+/−^ (*n*=13) embryos were observed to have straight tails compared to *cfap298^−/−^* (*n*=10), which displayed statistically significant greater tail curvature (for wild type and *cfap298*^+/−^, *P*≥0.9999; for wild type and *cfap298*^−/−^, *P*≤0.0001; for *cfap298*^+/−^ and *cfap298*^−/−^, *P*≤0.0001 by Fisher's exact test). However, for left–right defects, we did not observe a statistically significant difference between wild type, *cfap298*^+/−^ or *cfap298*^−/−^, but we did observe a statistically significant difference between *cfap298*^+/−^ and *cfap298*^−/−^ (for wild type and *cfap298*^+/−^, *P*=0.4091; for wild type and *cfap298*^−/−^, *P*=0.0705; for *cfap298*^+/−^ and *cfap298*^−/−^, *P*=0.0021 by Fisher's exact test). For embryos injected with 500 pg of wild-type *cfap298* mRNA, we observed no significant difference between wild-type (*n*=16), *cfap298*^+/−^(*n*=36) or *cfap298*^−/−^ (*n*=20) embryos (for wild type and *cfap298*^+/−^, *P*≥0.9999; for wild type and *cfap298*^−/−^, *P*≥0.9999; for *cfap298*^+/−^ and *cfap298*^−/−^, *P*≥0.9999 by Fisher's exact test) as well as no significant difference in left–right defects (for wild type and *cfap298*^+/−^, *P*=0.6769; for wild type and *cfap298*^−/−^, *P*≥0.9999; for *cfap298*^+/−^ and *cfap298*^−/−^, *P*≥0.9999 by Fisher's exact test). For embryos injected with 500 pg of *cfap298^ΔΔS^* mRNA, wild-type (*n*=23) and *cfap298*^+/−^(*n*=38) embryos were observed to have mostly straight tails compared to *cfap298*^−/−^ (*n*=27), which displayed statistically significant greater tail curvature (for wild type and *cfap298*^+/−^, *P*≥0.9999; for wild type and *cfap298*^−/−^, *P*≤0.0001; for *cfap298*^+/−^ and *cfap298*^−/−^, *P*≤0.0001 by Fisher's exact test) as well as significantly higher incidence in left–right defects (for wild type and *cfap298*^+/−^, *P*=0.2178; for wild type and *cfap298*^−/−^, *P*≤0.0001; for *cfap298*^+/−^ and *cfap298*^−/−^, *P*≤0.0001 by Fisher's exact test). We also observed a significant difference in tail curvature between *cfap298*^−/−^ uninjected, wild-type mRNA-injected and *ΔΔS* mRNA-injected embryos (for uninjected and wild-type mRNA injected, *P*≤0.0001; for uninjected and *ΔΔS* mRNA injected, *P*=0.0179; for wild-type mRNA injected and ΔΔS mRNA injected, *P*≤0.0001 by Fisher's exact test). We did not observe a significant difference in *cfap298*^−/−^ uninjected and *ΔΔS* mRNA-injected embryos (*P*=0.8864 by Fisher's exact test), but we did observe a significant difference in left–right defects in *cfap298*^−/−^ embryos injected with wild-type mRNA compared to uninjected embryos or embryos injected with *ΔΔS* mRNA (for uninjected and wild-type mRNA injected, *P*=0.0009; for wild-type mRNA injected and *ΔΔS* mRNA injected, *P*≤0.0001 by Fisher's exact test). *P<0.05, **P<0.01, ***P<0.001, ****P<0.0001. Scale bars: 500 μm.

Our results thus far indicate that amino acids Y161, D162 and P163 lie within a region of the mouse CFAP298 protein that functions in LR patterning and cilia motility. To determine if these residues perform a conserved function in LR asymmetry in vertebrates, we tested whether the *Cfap298^ΔΔS^* variant was capable of rescuing zebrafish *cfap298* null mutants. The *cfap298^tj271^* allele has previously been shown to be a loss-of-function mutation, and zebrafish embryos homozygous for *cfap298^tj271^* (hereafter referred to as *cfap298*^−/−^) display several phenotypes associated with cilia motility defects, including body and tail curvature, randomized heart jogging and kidney cysts (Jaffe et al., 2016) (Fig. 4A,C). Injection of *cfap298*^−/−^ embryos with 500 pg of wild-type zebrafish *cfap298* mRNA fully rescued both body curvature and LR defects, as determined by scoring heart jogging at 48 hpf (Fig. 4C,D). By contrast, injection of *cfap298*^−/−^ embryos with zebrafish *cfap298^ΔΔS^* mRNA, which contains the orthologous *ΔΔS* amino acid changes (ΔH161, ΔD162 and P163S), failed to rescue LR defects (Fig. 4B,C). Interestingly, 40% (11/27) of *cfap298^−/^*^−^ embryos injected with *cfap298^ΔΔS^* mRNA displayed normal body curvature, which was significantly higher than uninjected mutants, but still significantly lower than mutants injected with wild-type mRNA (Fig. 4C,D). Although the cellular basis of body curvature phenotypes in zebrafish cilia mutants is not fully understood (Bearce and Grimes, 2021), it appears that *cfap298^ΔΔS^* can partially execute its function in maintaining body and tail straightness, indicating that *Cfap298^ΔΔS^* is a partial loss-of-function mutation. Taken together, our evidence suggests that the mutation in *Cfap298^ΔΔS^* defines a three amino acid region crucial for cilia motility.

## DISCUSSION

The mechanisms underlying LR symmetry breaking are highly conserved across vertebrates, and the involvement of motile cilia in the process is well established. In previous work, we demonstrated an essential role for *Cfap298* in LR symmetry breaking in zebrafish through its function in cilia motility (Austin-Tse et al., 2013; Jaffe et al., 2016). Here, through the use of a previously unreported mutant allele, *Cfap298^ΔΔS^*, we reveal that *Cfap298* is also essential in LR axis formation in mice. Remarkably, changes in only three amino acids of the CFAP298 protein result in severe defects in LR organ positioning, preceded by abnormal LR patterning, as determined by *Nodal*, *Pitx2* and *Lefty1* expression in the LPM and midline. Given that ciliogenesis and planar polarization within the LRO are largely unaffected in *Cfap298^ΔΔS^* mutant embryos, we conclude that defects in LR symmetry breaking are due to a loss of cilia motility and fluid flow. Although we have not directly visualized cilia movement or fluid flow within the node, the immotility of cilia within the trachea of a *Cfap298^ΔΔS^* mutant is consistent with this interpretation. Further, the failure of the *Cfap298^ΔΔS^* variant to rescue LR patterning defects in zebrafish point to a conserved role for this region of the CFAP298 protein in cilia motility.

The *Cfap298^ΔΔS^* mutation affects a region of the protein that has not been previously implicated in the cilia motility function of *Cfap298*. The Chlamydomonas ortholog of *Cfap298*, *FBB18*, and the zebrafish ortholog, *kurly*, have been shown to interact with flagellar and motile cilia dynein arm components, as well as dynein arm assembly factors (Austin-Tse et al., 2013; Jaffe et al., 2016; Wang et al., 2022). Human pathogenic mutagenic alleles, which have been previously reported to truncate the CFAP298 protein near the C-terminal end of the protein, were also shown to disrupt dynein arm assembly (Austin-Tse et al., 2013; Wang et al., 2022). FBB18 interacts with chaperone proteins, and it has been posited that FBB18 is a co-chaperone for dynein assembly factors (Wang et al., 2022). Therefore, loss of cilia motility in *Cfap298^ΔΔS^* mutants are likely the result of lost or reduced dynein arm assembly. We hypothesize that amino acids Y161, D162 and P163S in CFAP298 may constitute an interaction site for components of the dynein assembly machinery or its chaperones. Alternatively, given we see a reduction in CFAP298 protein by western blot, mutation of these three amino acids may primarily affect stability and abundance of the protein. This would suggest that there are differential thresholds of CFAP298 protein necessary for robust cilia motility and maintaining proper axis straightness in our zebrafish assay. Future experiments focused on determining if CFAP298 protein–protein interactions are altered by the ΔΔS mutation may provide evidence needed to evaluate which model is correct.

When CFAP298 was initially annotated, it was described as consisting of two domains: a coiled-coil domain and a domain of unknown function (DUF) (Austin-Tse et al., 2013). Recently, the crystal structure of the Chlamydomonas ortholog FBB18 was solved, showing it consists of a ubiquitin-like (UBL) domain and a so-called loop domain (Yamamoto et al., 2025). In relation to these domains, the *Cfap298^ΔΔS^* mutation is predicted to affect a short alpha-helix located in the linker between loop and UBL domains. How this region could be working in conjunction with the UBL and loop domain of CFAP298 in dynein arm assembly also warrants future investigation.

Planar polarization of nodal cilia is important for directed cilia motility and the production of leftward fluid flow (Hashimoto et al., 2010; Song et al., 2010), and several pieces of evidence had previously implicated CFAP298 in PCP establishment. In the mouse epidermis, which is strongly planar polarized but lacks motile cilia, we did not observe defects in PCP establishment in *Cfap298^ΔΔS^* mutants, nor did we observe changes in PCP asymmetry or planar polarized basal body positioning in cells with motile cilia (the node). This supports our conclusion that the ΔΔS mutation selectively impairs its cilia motility-related function. It is possible that other regions of the CFAP298 protein are important for PCP establishment in mice. Further investigations using a loss-of-function allele would be required to determine the function of *Cfap298* in planar polarization.

## MATERIALS AND METHODS

### Generation of the *Cfap298^ΔΔS^* mouse line, mouse husbandry and breeding

*Cfap298* deletion mutations were generated using CRISPR-Cas9. The CRISPR target sequence was TGCCACCATACGATCCTATCCGG (PAM site underlined) in exon 4. Tracr and crRNA (Sigma) were prepared with Cas9 protein (Sigma) and microinjected into C57BL/6J zygotes. Founders were determined to have insertions or deletions by Sanger sequencing and PCR (Genewiz). One founder was observed to have a 6 bp deletion which removed two amino acids and changed proline 163 to serine. The founder was backcrossed to C57BL/6J mice once and then bred into a C3H/HeJ background. *Cfap298^ΔΔS^* carrying mice were outcrossed into the C3H/HeJ background for a minimum of 10 generations and transmission of the mutation was determined by PCR. For PCRs, the forward primer sequence was CCCAATGCACTTTCAGAAACA and the reverse sequence was ACCCTGCCCCACATACCT. Genotyping PCR produces two bands for a heterozygous mouse with the *Cfap298^ΔΔS^* amplicon being six bases smaller than the wild type. To generate *Cfap298^ΔΔS^*^/*ΔΔS*^ homozygous embryos, *Cfap298^ΔΔS^*^/+^ adult mice were mated. Staging of embryos was estimated based on the time a mating plug was observed (E0.5). *Cfap298^ΔΔS^* homozygous embryos were identified by a PCR showing only the mutant amplicon. All mice used for obtaining embryos were at least 6–8 weeks of age.

All animal procedures were approved by Princeton's Institutional Animal Care and Use Committee (IACUC). All mice were housed in facilities accredited through the American Association for Accreditation of Laboratory Animal Care (AAALAC).

### E13.5–E15.5 embryo and adult necropsies

For embryo necropsies, E13.5–E15.5 embryos were dissected in PBS. Tissue from tails were collected for genotyping. Whole embryos with incisions on the sides of the abdomen were then incubated in 4% PFA/PBS overnight at 4°C to allow for fixation of internal organs. Embryos were washed twice in PBS. For postnatal stages, pups were ear punched 12 days after birth and genotyped for *Cfap298^ΔΔS^*. For adult necropsies, mice were euthanized under CO_2_. Body cavities were opened to determine situs.

### E8.5 embryo hybridization chain reaction

The hybridization chain reaction (HCR) procedure was adapted from Matthew Anderson and Mark Lewandoski, NIH, modified from Choi et al. (2020). E8.5 embryos were dissected in ice-cold 1×PBS and then fixed overnight at 4°C in 4% PFA/PBS. Embryos were then washed twice for 5 min in PBS with 0.1% Tween20 (PBSTw), followed by a series of dehydration steps into 100% methanol. Embryos were incubated in methanol for at least 2 h or overnight before proceeding with the probe step. Embryos were rehydrated into 100% PBSTw and then bleached in 6% hydrogen peroxide in PBS for 20 min. Embryos were washed twice for 5 min in PBSTw while rocking. Embryos were then treated in a 1:1 mix of 10 μg/ml proteinase K in PBSTw for 1 min then washed twice for 5 min in PBSTw. The embryos were fixed again in 4% PFA for 20 min at room temp while rocking. Embryos were washed three times for 5 min in PBSTw, transitioned into 1:1 hybridization buffer warmed to 37°C and PBSTw for 10 min while rocking. Embryos washed for 10 min at room temperature in prewarmed hybridization buffer. Hybridization buffer was replaced with fresh buffer and embryos were incubated in hybridization chamber at 37°C while rocking for 1–3 h. Embryos were then incubated in probe+hybridization buffer solution overnight in chamber. Embryos were washed in probe wash buffer warmed to 37°C three times for 20 min at 37°C while rocking. Embryos were rinsed in 5× saline sodium citrate with 0.1% Tween 20 (SSCT) three times for 5 min at room temperature while rocking. Embryos were then washed in 1:1 amplification buffer and PBSTw for 10 min at room temperature while rocking and then transferred into amplification buffer for 30 min at room temperature while rocking. Embryos were then incubated in hairpins prepared in amplification buffer overnight at room temperature away from light. One quick wash in 5×SSCT was followed by three 5 min washed in 5×SSCT at room temperature while rocking and away from light. Embryos were then whole mounted in SlowFade Glass Soft-Set Antifade mounting media. After imaging, embryos were lysed in 95°C 50 mM NaOH for genotyping. Embryos were imaged on Nikon A1R-Si confocal microscopes. Images acquired were processed using NIS elements, ImageJ and Adobe Photoshop.

### Western blot analysis

To validate anti-CFAP298 antibodies, mouse keratinocytes derived from CD1 pups were cultured using E media (Nowak and Fuchs, 2009) supplemented with 15% fetal bovine serum and 50 μM calcium. Keratinocytes were then transfected with either Cfap298-ALFA tagged or ALFA tag only constructs (Billie. M. Reneker, Princeton University; unpublished). Two days following transfection, cells were lysed in 1 ml TE lysis buffer [1% Triton X-100, EDTA (pH 8) 0.5 M, 1 Pierce protease inhibitor tablet] on ice and centrifuged for 15 min at 4°C at 8000 ***g***. Supernatants were collected and run on a 10% SDS-PAGE gel.

For embryonic tissue lysates, E15.5 embryos were dissected in PBS. Tails were collected for genotyping. Heads and limbs were removed, flash frozen in liquid nitrogen, then stored at −80°C. Tissue samples were then ground using a cryomill. Samples were then lysed in 700 μl TE lysis buffer, incubated on ice for 15 min, then vortexed, centrifuged for 15 min at 17,000 ***g*** at 4°C and supernatants collected.

Samples were run on 10% SDS-PAGE gel, transferred onto a nitrocellulose membrane and incubated with anti-Cfap298 rabbit (1:250; Invitrogen, PA5-53803) and FluoTag-X2 anti-ALFA fluorescently tagged with LI-CORDye 680RD (1:500; Nanotag, N1502-Li800-L) primary antibodies diluted in 5% BSA in PBS with 0.05% Tween 20 overnight at 4°C. Membranes were washed three times for 15 min and incubated with IRDye800CW goat anti-rabbit-IgG (LI-COR, 926-32211) secondary antibody. Following mild stripping, membranes were incubated with anti-β-tubulin rat primary (1:10,000; Abcam, ab6160) and IRDye800CW goat anti-rat-IgG secondary (1:10,000; LI-COR, 926-32219) antibodies. Uncropped western blot images are shown in Fig. S5.

### Immunofluorescence

For immunofluorescent labeling of the node, we followed an immunostaining protocol adapted from Sai et al. (2022). E8.5 embryos were dissected in ice-cold PBS then fixed in 4% PFA in PBS on ice for 45 min. Embryos were washed three times for 10 min in PBS+0.2% Triton X-100 (PBST) then incubated in blocking solution (1% bovine serum albumin and 2.5% normal donkey serum prepared in PBST) for 1 h at room temperature. After blocking, embryos were incubated in primary antibodies diluted in blocking solution overnight at 4°C. Following primary antibody incubation, embryos were washed three times for 15 min in PBST, followed by incubation in secondary antibodies and Hoechst 33342 prepared in PBST overnight at 4°C. Embryos were then washed three times for 5 min in PBS and wholemounted in SlowFade Glass Soft-Set Antifade mounting media. After imaging, embryos were lysed in 95°C 50 mM NaOH for genotyping.

For immunostaining of mouse embryonic skins, tails were first removed from E15.5 embryos for genotyping followed by fixation in 4% PFA/PBS with Mg^2+^ and Ca^2+^ for 1 h at room temperature while rocking. Skins were then dissected from embryos and washed three times in PBST for 5 min each followed by incubation in blocking solution (PBST+1% fish gelatin, 1% bovine serum albumin and 2.5% normal donkey serum) for 1 h at room temperature while rocking. Skins were then incubated overnight in primary antibody solution prepared in blocking solution overnight at 4°C while rocking. Skins were then washed three times in PBST for 30 min each. Washed skins were then incubated in secondary antibody and Hoechst solution prepared in PBST overnight at 4°C while rocking. Skins were washed three times for 10 min each followed by two 5 min washes in PBS. Skins were mounted epidermal side up in Prolong Gold curing mounting media and imaged after curing.

Embryos and embryonic tissues were imaged on a Nikon A1R-Si confocal microscope. Images were processed using NIS elements, ImageJ and Adobe Photoshop.

The following primary and secondary antibodies used: guinea pig anti-Celsr1 (1:1000; Devenport and Fuchs, 2008), mouse anti-acetylated-tubulin (1:200; Sigma, T6793), mouse anti-γ-tubulin (1:200; Sigma, T6557), rat anti-P-cadherin (1:250; Invitrogen, 13-2000z), rabbit anti-Sox9 (1:1000; Millipore, AB5535), Alexa Fluor 488 donkey anti-guinea pig (1:1000; Jackson Immuno, 706-545-148). Anti-acetylated-tubulin primary antibody was directly conjugated with Alexa 555 fluorophore using Biotium Mix-n-Stain CF Dye Antibody Labeling Kit (92274), and anti-gamma-tubulin primary antibody was conjugated with Alexa 647 fluorophore using Biotium Mix-n-Stain CF Dye Antibody Labeling Kit (92274). For nuclear staining, Hoechst dye was diluted to 1 μg/ml.

### Quantification of planar cell polarity in the node and skin

Celsr1 images were used to generate segmentation masks using Cell Pose software (Stringer et al., 2021) followed by further hand corrections of segmented masks using TissueAnalyzer software for FIJI (Aigouy et al., 2010). Polarity analysis was determined as previously described (Aigouy et al., 2010; Aw et al., 2016). Segmented masks were used in TissueAnalyzer to generate the axis and magnitude of Celsr1 polarity. Rose plots were then generated for Celsr1 polarity using MATLAB. The average polarity magnitude data was exported for statistical analysis in GraphPad Prism 10.

### Live imaging trachea MCCs

Tracheas were removed from euthanized adult mice. Preparation of trachea for live imaging was performed as previously described (Francis and Lo, 2013). Dissected tracheas were cut in half and placed lumen side down onto a glass-bottomed dish and covered in ∼100 μl of PBS. A square was cut into a circular piece of parafilm and another circular piece of parafilm was adhered to the first piece using nail polish to create a small chamber. This was placed over lumens in the imaging dish. Tracheas were live imaged on a Nikon Eclipse Ti2 equipped with an ORCA-Fusion BT Hamamatsu digital camera.

### Quantification of nodal cilia length and basal body displacement

Nodal cilia were labeled with anti-acetylated α-tubulin and basal bodies were visualized with gamma tubulin. *Z*-stacks encompassing the full node depth were collected at 1 μm optical steps. Stacks were processed in ImageJ and analyzed with CiliaQ (Hansen et al., 2021; Schindelin et al., 2012). For each nodal cell the apical boundary (Celsr1) was segmented and the centroid of each node cell recorded. The gamma tubulin position relative to the centroid along the anterior posterior axis was then determined. Basal bodies were classified as anterior, posterior or central/equal. Quantitative data were exported for statistical analysis and plotting in GraphPad Prism 10.

### Zebrafish strain maintenance

The zebrafish *cfap298^tj271^* mutant line, which has been previously described (Jaffe et al., 2016), was maintained by outcrossing with wild type (Burdine lab strain PWT). Transmission of the mutation was determined by PCR and BSTN1 restriction enzyme digest, as described previously (Jaffe et al., 2016). To generate *cfap298^tj271^*^/*tj271*^ (*cfap298*^−/−^) homozygous embryos, *cfap298^tj271^*^/+^ fish were crossed. Embryos were maintained at 28°C in E3 (5 mM NaCl, 0.17 mM KCl and 0.33 mM CaCl_2_). All zebrafish used for experiments were at least 6 months old.

All animal procedures were approved by Princeton's Institutional Animal Care and Use Committee (IACUC).

### Zebrafish mRNA injection and quantification of phenotypic rescue

mRNA for injections was prepared as previously described (Jaffe et al., 2016). Wild-type *cfap298* mRNA was synthesized from a pSport6.1 vector using a Sp6 mMessage Machine *in vitro* transcription kit (Thermo), while the *cfap298^ΔΔS^* mRNA was synthesized from a pBluescript vector using a T7 mMessage mMachine (Thermo) *in vitro* transcription kit. Embryos from a *cfap298^tj271^*^/+^ heterozygous cross were injected at the one-cell stage with 500 pg of mRNA. Body curvature and heart jogging were scored at 48 hpf. Embryos were then lysed in 50 mM NaOH boiled at 95°C and genotyped. Embryos were imaged using a Leica M205FA microscope.

## Supplementary Material

10.1242/joces.264129_sup1 Supplementary information
