# Supplementary material for: A conserved domain of Cfap298 governs left–right symmetry breaking in vertebrates
Source: J Cell Sci. 2025 Oct 31;138(20):jcs264129. doi: 10.1242/jcs.264129 (PMC12633728; doi:10.1242/jcs.264129)
Supplement: Supplementary information [file joces-138-264129-s1.pdf]

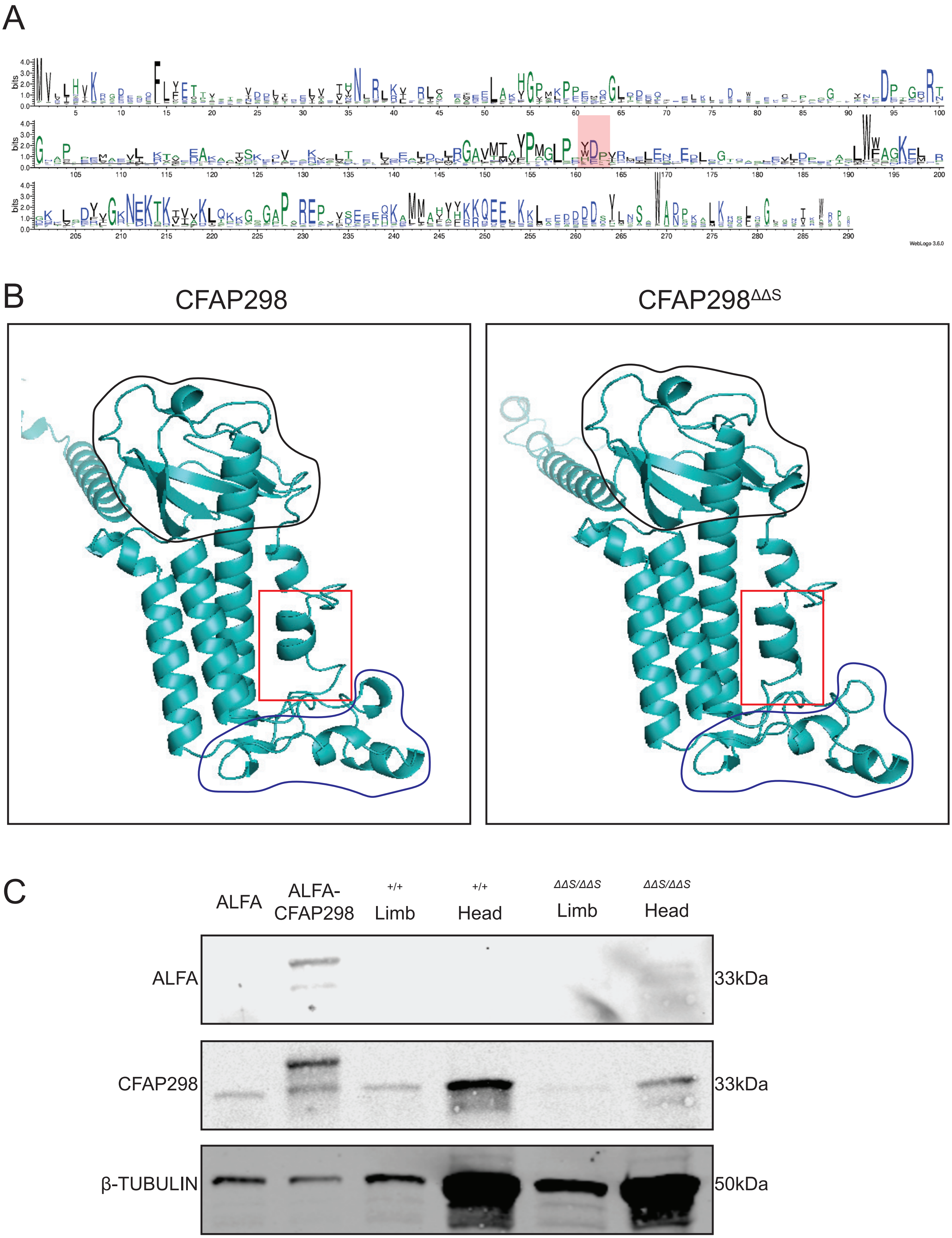

Journal of Cell Science • Supplementary information

Fig. S1. ΔΔS regions is highly conserved and is important for stability. A) DMfold multiple sequence alignment logo of CFAP298 from 1610 species. Sequence alignment shows frequency of an amino acid at a given location along the 290 amino acids of CFAP298. Size of the amino acid at a position indicates high incidence of that amino acid at that site suggesting higher conservation. Amino acids 161-163 (orange box) show high conservation across species. B) Alphafold predicted structures of wildtype CFAP298 and CFAP298$^{\Delta\Delta S}$ proteins. Red box indicates the region that includes amino acids 161-163. Black outline indicates the location of the Ubiquitin-like domain . Blue outline indicates location of the Loop domain. C) Western blot to detect CFAP298 protein. Limb and head lysates from wild-type and *Cfap298*$^{\Delta\Delta S}$ mutant E15.5 embryos were probed with anti-CFAP298 antibodies (middle row, right columns). Lysates from keratinocytes transfected with ALFA tag only or *Cfap298*-ALFA were used to test specificity of the CFAP298 antibody (left colums).

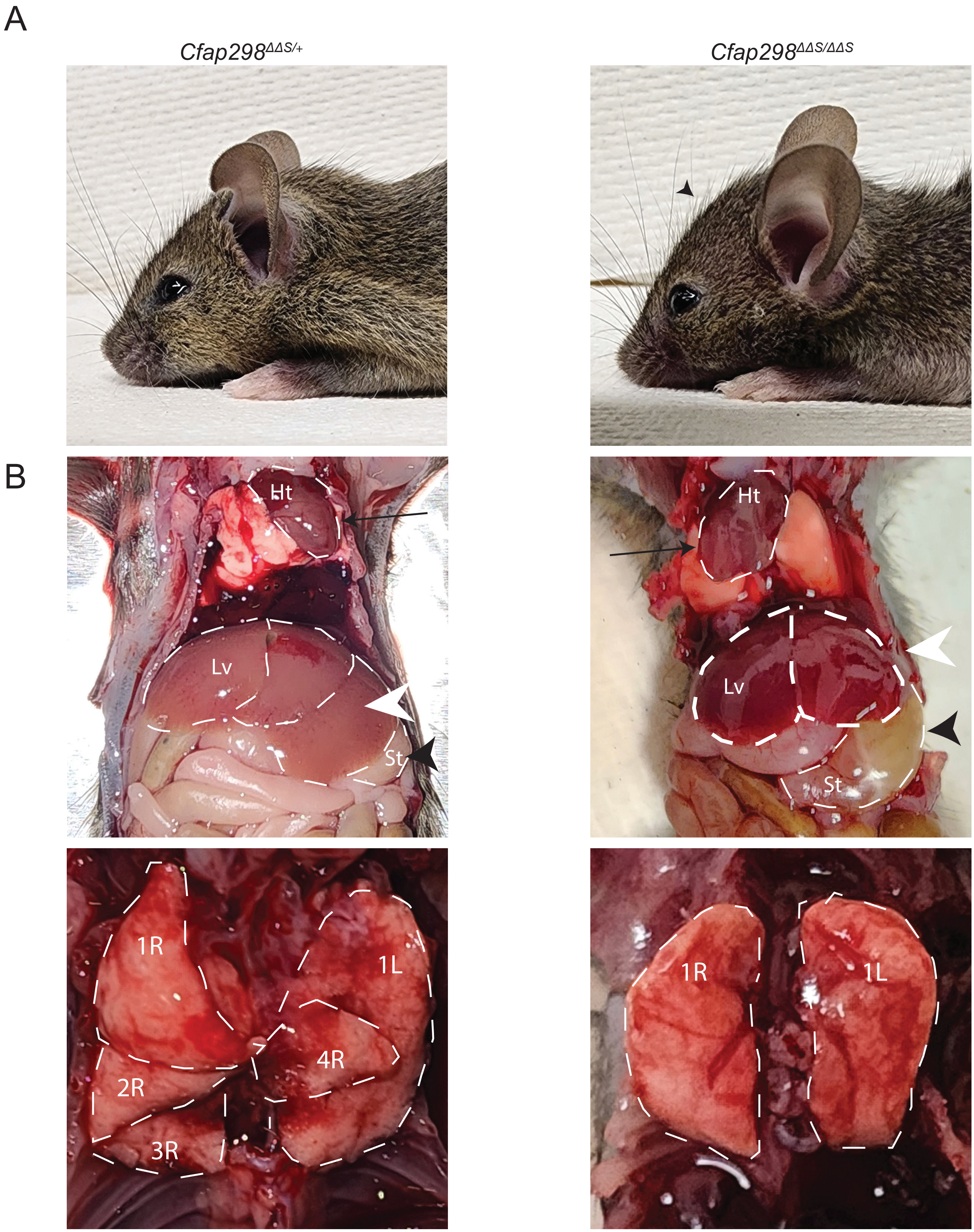
A
$Cfap298^{\Delta\Delta S/+}$
$Cfap298^{\Delta\Delta S/\Delta\Delta S}$
B
Ht
Lv
St
Ht
Lv
St
1R
1L
2R
3R
4R
1R
1L

Fig. S2. Adult *Cfap298*$^{\Delta\Delta S}$ mutant displays cilia motility related defects. A) Side view of *Cfap298*$^{\Delta\Delta S}$ heterozygote and homozygous adult mice. Mutant adult shows expansion and doming of the head (arrowhead). B) Representative images of adult necropsies showing left-right defects. *Cfap298*$^{\Delta\Delta S/+}$ adult has left-sided heart, three-lobed liver and left sided stomach. Lungs from *Cfap298*$^{\Delta\Delta S/+}$ display 4 lobes on the right and 1 on the left. *Cfap298*$^{\Delta\Delta S}$ mutant images showing right-sided heart, two-lobed liver, and left-sided stomach. Mutant adult also displays left lung isomerism. Heart (Ht, arrow), liver lobation (Lv, white arrowhead), and stomachs (St, black arrowhead) are outlined. Individual lung lobes are outlined and labeled with their position on either left (L) or right (R).

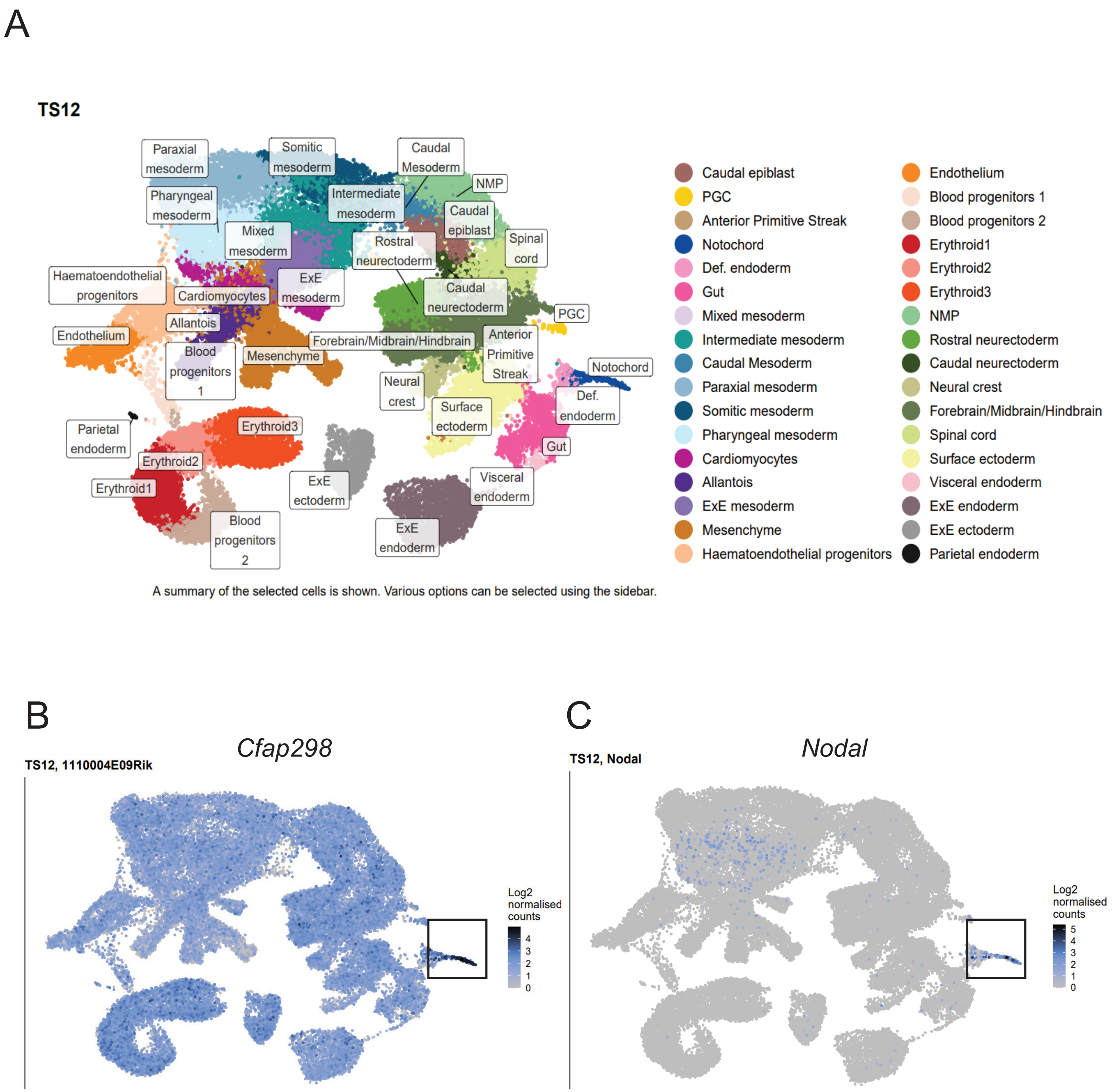

Fig. S3. Single- cell RNA sequencing analysis of *Cfap298* expression shows enrichment in the mouse Left-Right Organizer. A) UMAP displaying scRNA seq data from E8.5 embryos (Pijuan-Sala et al., 2019). B) *Cfap298* is expressed in most E8.5 cell clusters and appears to be significantly enriched in the notochord/node cluster (boxed region). C) *Nodal* is specifically expressed within the notochord/node (boxed region) and mesoderm.

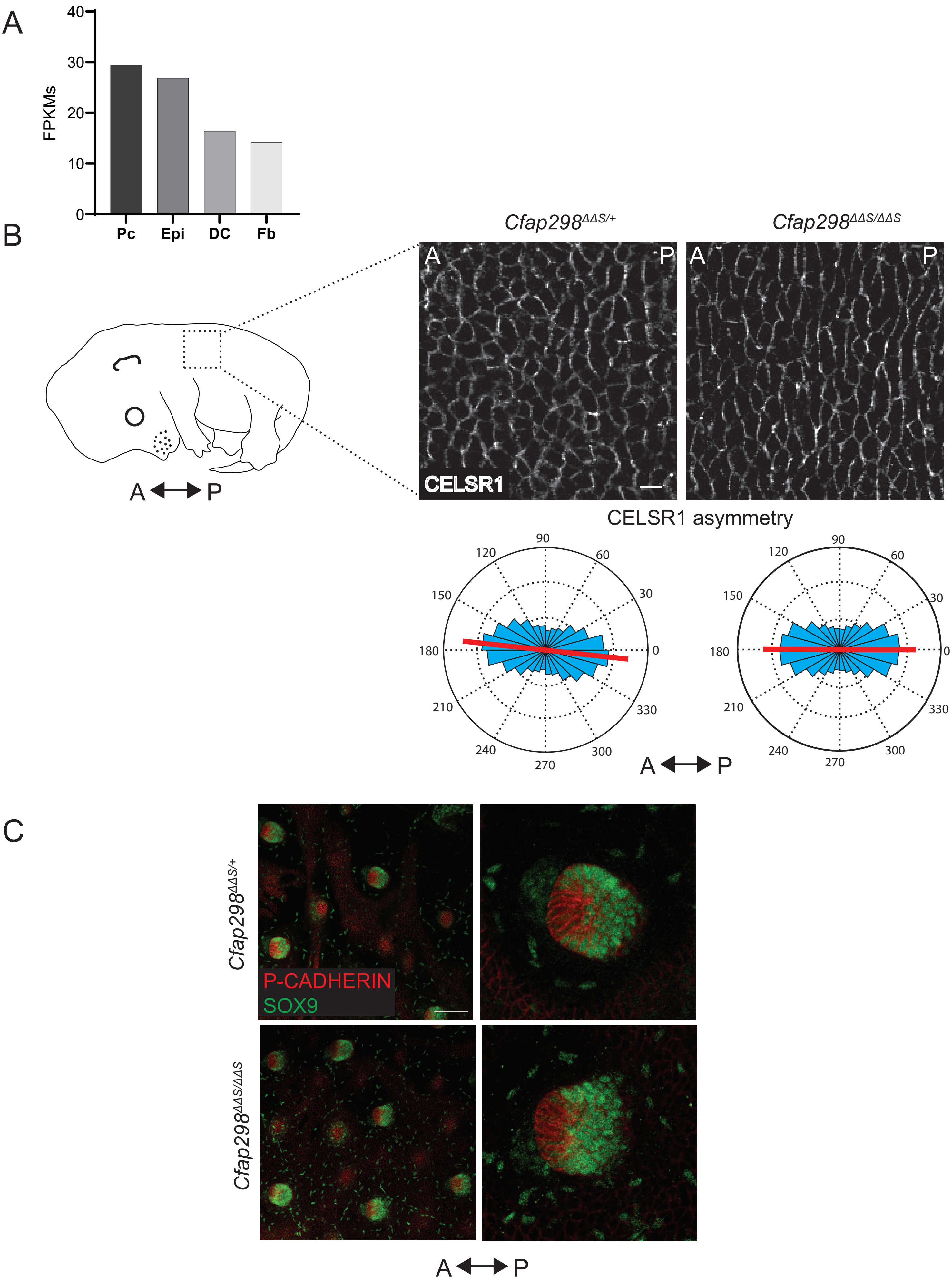

Journal of Cell Science • Supplementary information

Fig. S4. Planar polarity in the mouse epidermis is unaffected in *Cfap298*^{ΔΔS} mutants. A) Bulk RNA seq data from E14.5 embryonic skin from (Sennet el al., 2015; Rezza et al., 2016) showing *Cfap298* expression in the placode (Pc), the epidermis (Epi), dermal condensate (DC), and dermal fibroblasts (Fb). B) Schematic showing region of epidermal skin dissected for CELSR1 staining and quantification in the skin with the anterior and posterior axis (AP) marked along the embryo. Planar view of the basal layer of the epidermis showing Celsr1 staining (grayscale) from E15.5 *Cfap298*$^{ΔΔS/+}$ and *Cfap298*^{ΔΔS} mutant embryos. Circular histograms depict magnitude and angle of CELSR1 polarity relative to the embryonic anterior-posterior axis for *Cfap298*$^{ΔΔS/+}$ (n=3, 11,972 cells total) and *Cfap298*^{ΔΔS} mutant (n=3 embryos, 12,313 cells total) embryos. Scale bar= 10um. C) Images of hair follicles from *Cfap298*$^{ΔΔS/+}$ and *Cfap298*^{ΔΔS} mutant skins with anterior cells marked by P-CADHERIN (red) and posterior cells marked by SOX9 (green). Scale bar=100um.

Journal of Cell Science • Supplementary information

A

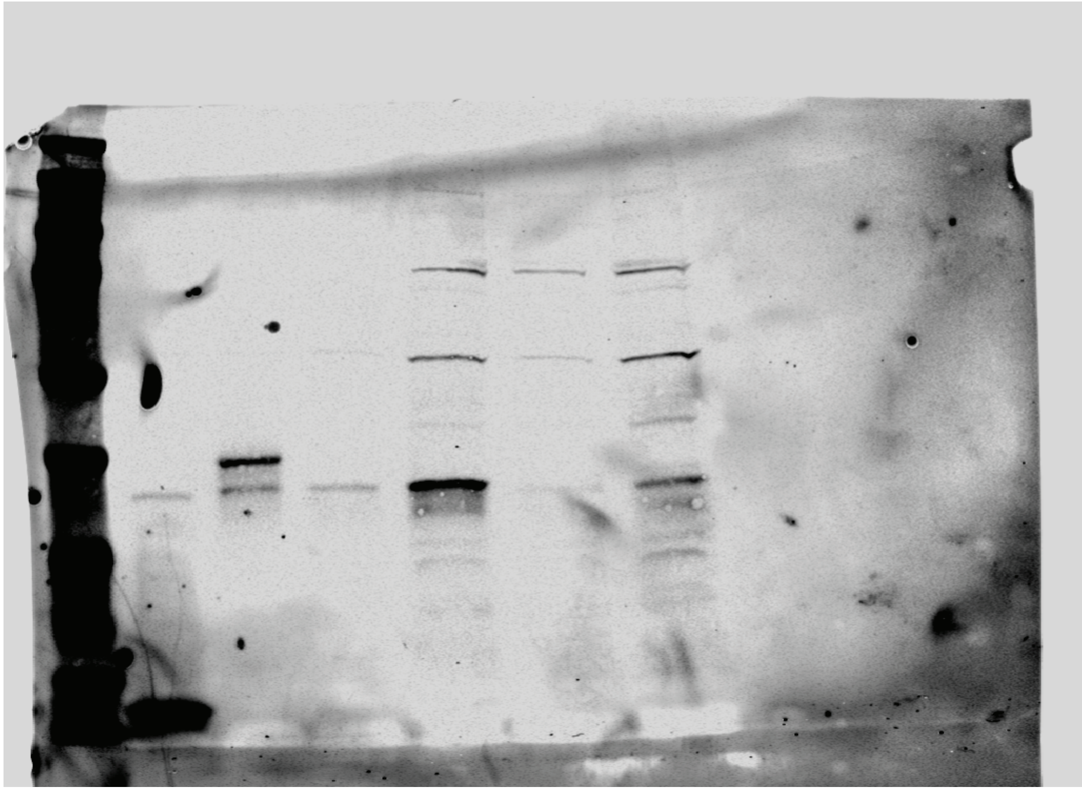

B

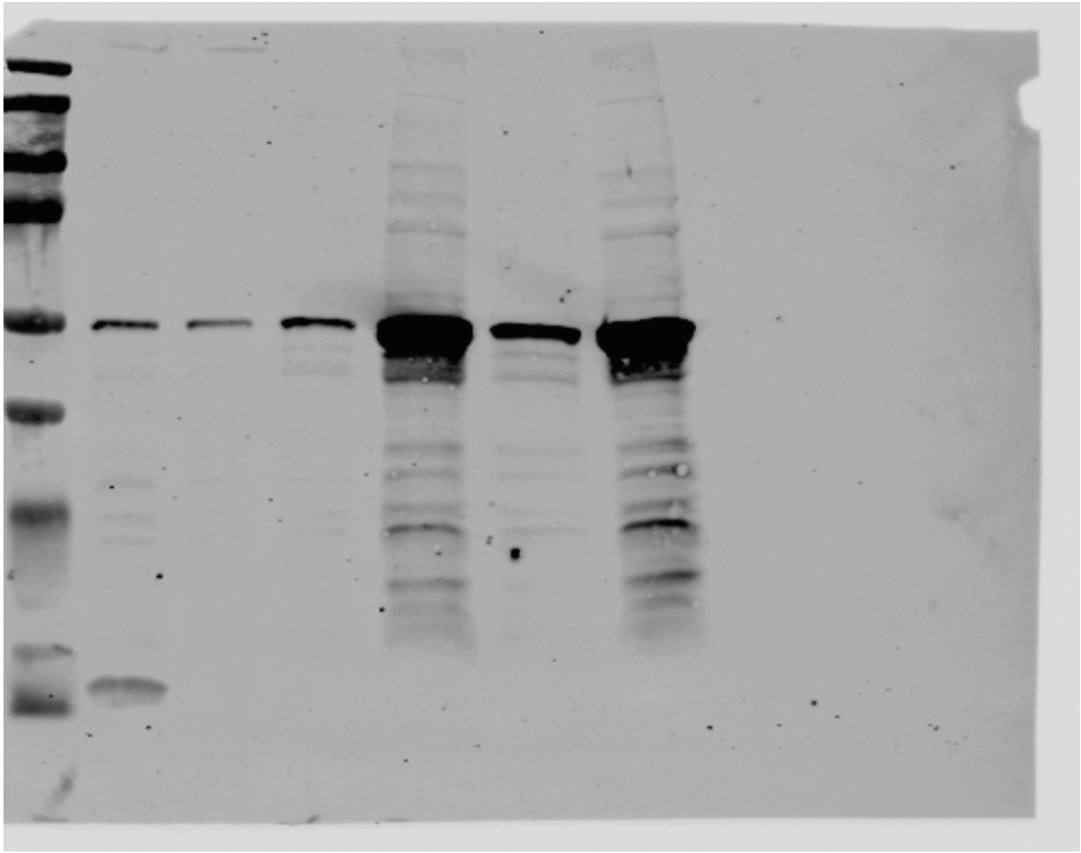

Fig. S5. Uncropped Western blot corresponding to Supplemental figure 1C.
A) Uncropped Western blot stained with CFAP298 and ALFA antibodies. B) Western blot from A) stripped and re-probed with Beta-TUBULIN antibody.

Journal of Cell Science • Supplementary information

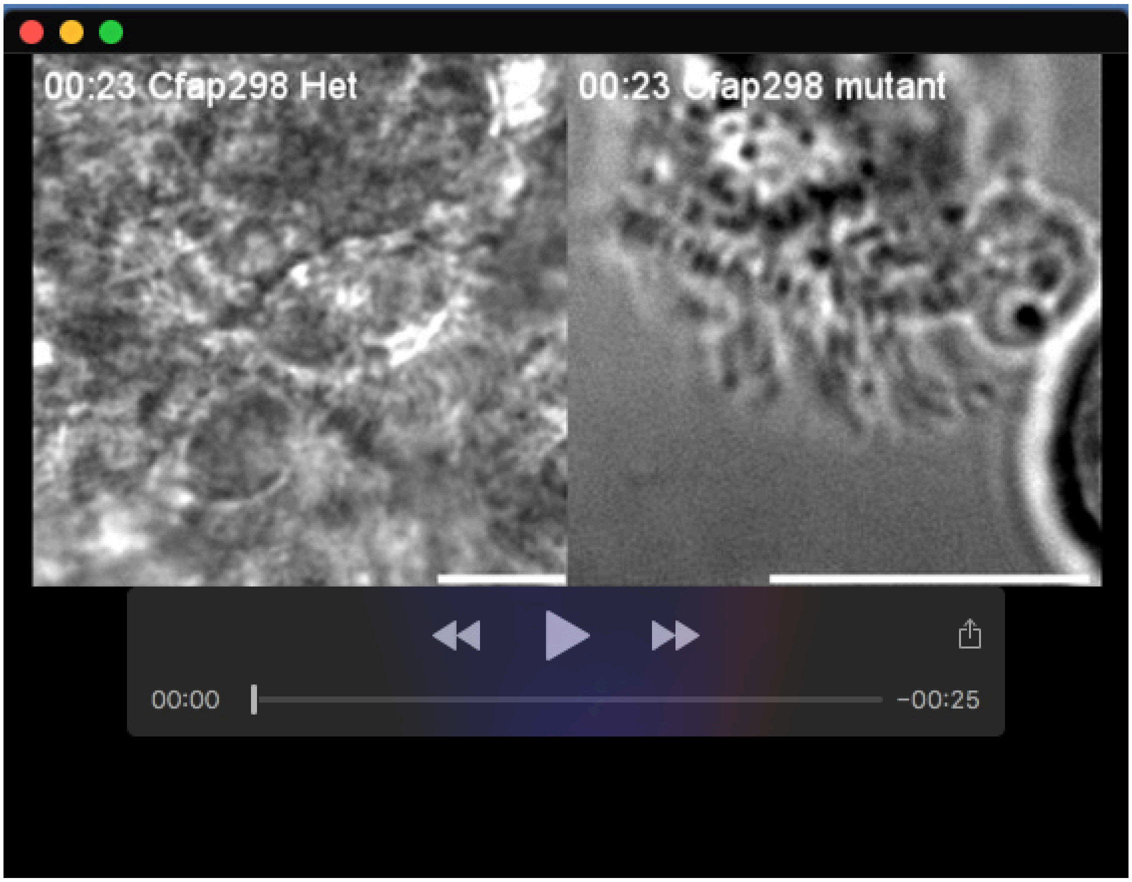

Movie 1. Planar view of MCCs from *Cfap298*$^{\Delta\Delta S/+}$ and *Cfap298*$^{\Delta\Delta S}$ mutant tracheas.

Videos showing planar views of motile cilia from *Cfap298*$^{\Delta\Delta S/+}$ tracheas and immotile cilia in *Cfap298*$^{\Delta\Delta S}$ mutant tracheas. Videos were taken at 7fps. Scale bar= 20um.

Journal of Cell Science • Supplementary information

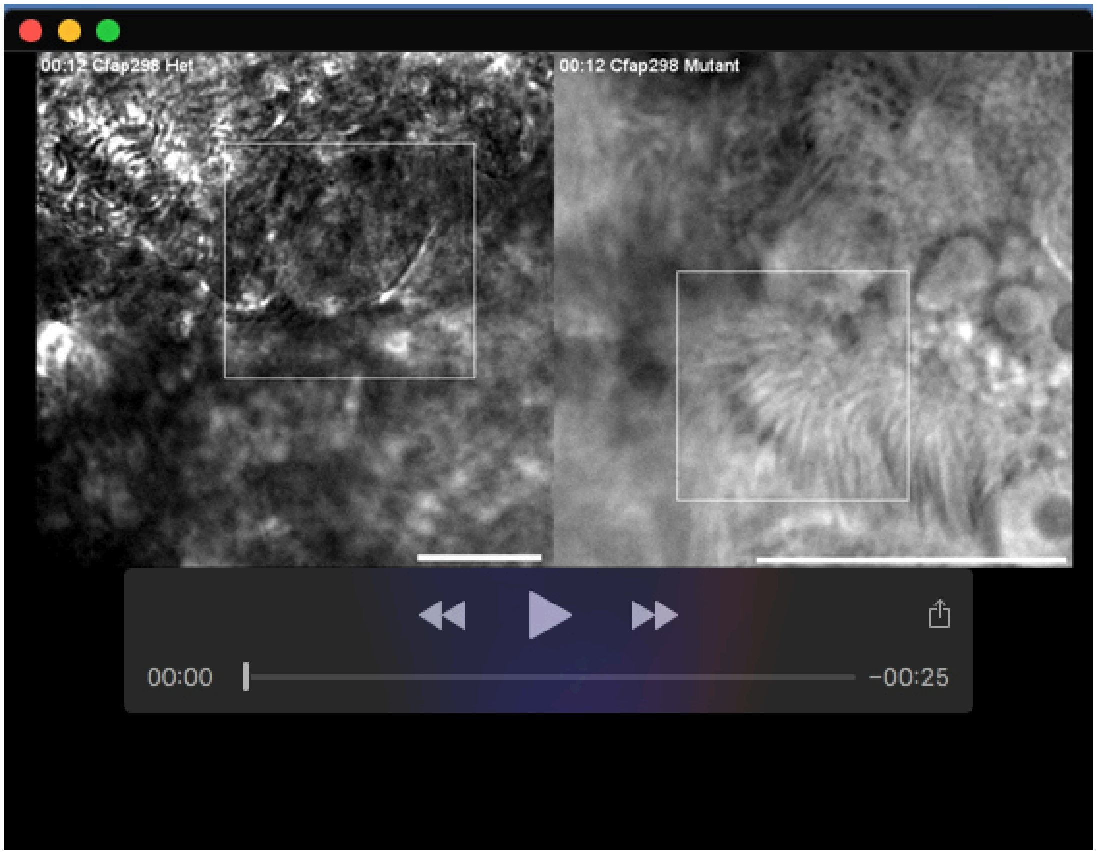

Movie 2. Side view of MCCs from *Cfap298*$^{\Delta\Delta S/+}$ and *Cfap298*$^{\Delta\Delta S}$ mutant tracheas.

Videos showing side view of motile cilia from *Cfap298*$^{\Delta\Delta S/+}$ tracheas and immotile cilia in *Cfap298*$^{\Delta\Delta S}$ mutant tracheas. Videos were taken at 7fps. Scale bar=10um.

Journal of Cell Science • Supplementary information
